# Detection and Molecular Characterization of Animal Adenovirus and Astrovirus from Western Maharashtra, India

**DOI:** 10.3390/v15081679

**Published:** 2023-08-01

**Authors:** Pradeep M. Sawant, Rishabh B. Waghchaure, Pooja A. Shinde, Avani P. Palikondawar, Mallika Lavania

**Affiliations:** Enteric Viruses Group, ICMR—National Institute of Virology, 20-A, Ambedkar Road, Pune 411 001, India; rbwaghchaure1997@gmail.com (R.B.W.); poojashinde483@gmail.com (P.A.S.); avanipalkratwar@gmail.com (A.P.P.); mallikalavania@gmail.com (M.L.)

**Keywords:** molecular detection, characterization, astrovirus, adenovirus, animal

## Abstract

Astroviruses (AstV) and adenoviruses (AdV) are associated with diarrhoea in young animals. However, the epidemiology and genetic diversity of AstVs and AdVs in animals is not well studied. Hence, the present study was conducted to detect and characterize AstVs and AdVs in calves, piglets and puppies from Western Maharashtra, India. Out of the processed porcine (48), canine (80), and bovine (65) faecal samples, the porcine AstV (PAstV), bovine AstV (BAstV), canine AstV (CAstV), and porcine AdV (PAdV) were detected in 12.5%, 7.69%, 3.75% and 4.1% of samples, respectively. In the RNA-dependent RNA polymerase region-based phylogenetic analysis, the detected BAstV strains grouped with MAstV-28, MAstV-33, and MAstV-35, CAstV strains belonged to MAstV-5; PAstV strains belonged to MAstV-24, MAstV-26, and MAstV-31. However, in hexon gene-based phylogeny, both the detected PAdV were of genotype 3, exhibiting 91.9–92.5% nucleotide identity with Ivoirian and Chinese strains. The study reports first-time BAstVs from calves and PAdV-3 from piglets in India. The study revealed diversity in the circulation of AstVs in tested animals and AdVs in pigs, and suggested that they alone might be associated with other diarrhoea or in combination with other enteric pathogens, thus highlighting the necessity of extensive epidemiological investigations to develop diagnostic tools and control measures.

## 1. Introduction

Diarrhoea is the leading infectious cause of death among neonates of animals. The farmers and pet owners suffer a huge economic loss from diarrheal disease. Therefore, monitoring of diarrhoeal diseases should be performed on a priority basis. Acute diarrhoea is caused predominantly by rotavirus and norovirus worldwide. Some countries have introduced rotavirus vaccines in animals to provide effective protection. However, studies on other diarrheal viruses such as adenoviruses (AdVs) and astroviruses (AstVs) have been largely neglected [1,2,3].

AdVs are double-stranded DNA viruses belonging to the family *Adenoviridae*. The AdV genome consists of the origin of replication at both ends (inverted terminal repeats), viral packaging sequences at the left-hand side of the genome, five early transcription units (E1-E4), intermediate transcription units (IX, IVa2) and major late transcripts (code for structural proteins). All the member genera have similar structures and genus-specific genes; Mastadenoviruses contain (protein IX, protein V, and a few proteins encoded by E1, E3 and E4 regions), while Atadenovirus contain (p32, LH3) but lack gene coding for protein IX and V) [3]. Of the five serotypes of porcine AdV (PAdV) belonging to Genus Mastadenovirus, PAdV-1-3, PAdV-4 and PAdV-5, they correspond to three species PAdV-A, PAdV-B, and PAdV-C, respectively. Additionally, two PAdV serotypes have also been proposed [4]. Although PAdVs rarely cause diseases of economic concern in the domestic population. In deceased pigs, PAdVs are found to be associated with certain diseases such as encephalitis, nephritis, respiratory diseases and reproductive disorders [5]. The bovine AdV (BAdV) belong to the genera, *Mastadenovirus* and *Atadenovirus*, with 10 recognized serotypes. The *Mastadenovirus* genus contains BAdV-1 (BAdV-A), BAdV-2 (Ovine adenovirus-B), BAdV-3 (BAdV-B), BAdV-9 (HAdV-C), BAdV-10 (BAdV-C) serotypes, respectively, whereas the serotypes BAdV-4, -5, -6, -7 and -8 are included in *Atadenovirus* genus as BAdV-D [4]. The majority of these serotypes cause mild diseases of the gastrointestinal or respiratory tract in the bovines [3]. Canine adenovirus (CAdV) belongs to the genus *Mastadenovirus*, divided into two serotypes: CAdV-1 and CAdV-2 [4]. The CAdV-1 type causes infectious canine hepatitis, which is life-threatening in puppies and encephalitis in foxes, racoons, bears and skunks. The CAdV-2 type is a cause of kennel cough in breeding dogs [3].

AstVs belonging to the family *Astroviridae* are non-enveloped, with single-stranded RNA as a genome arranged in three open reading frames; ORF1a and ORF1b codes for serine protease and RNA-dependent RNA polymerase (RdRp), and ORF2 codes for capsid proteins. The family consist of two genera, *Mamastrovirus* which includes 19 species, and *Avastrovirus*, which includes 3 species. The porcine AstVs (PAstVs) group contains five lineages, PAstV1-5, which have been classified into seven genotypes; MAstV-3, MAstV-22, MAstV-24, MAstV-26-27, MAstV-31-32. The bovine AstVs (BAstVs) are divided into seven genotypes MAstV-13, MAstV-28-30, and MAstV-33-35, while CAstVs are classified into MAstV-5 [6,7]. The high genetic diversity and widespread presence of AstV make diagnosis and control strategies extremely difficult. Although AstV is found in the faeces of animals with and without diarrhoea, most studies reported that the detection rate with symptoms of gastroenteritis is higher than a symptomatic one, indicating its potential role in diarrhoea. 

The characterization of animal AstV and AdV is of immense importance as India ranks first in cattle and buffalo population; pig farming is emerging as a lucrative business, and close contact with stray and pet dogs [8]. However, surveillance and genetic characterization studies for animal AstV and AdV in India are limited. Hence, the present study aimed at the detection and characterization of adenovirus and astrovirus from animals revealed previously undetected genotypes of BAstV, CAstV and PAdV.

## 2. Materials and Methods 

### 2.1. Sample Collection

The faecal samples were randomly collected from diarrheic and asymptomatic calves, piglets and puppies under six months of age between the 2017 to 2019 period from Western Maharashtra, India. The samples were transported on ice, stored at −80 °C until further processing, and were processed in batches of fifteen at a time. A 30% stool suspension was centrifuged at 10,000 rpm for 10 min, and the supernatant was collected and immediately processed for nucleic acid extraction. The bovine samples were already screened for RVA by RT-PCR [9,10] while porcine and canine samples were screened using published VP6 primers [9]. 

### 2.2. Nucleic Acid Extraction 

Total RNA and DNA were extracted from 140 µL of stool suspension using an RNA extraction kit (Qiagen, Germany), following the manufacturer’s recommendation. The extracted nucleic acids were stored at −80 °C until their use as a template in reverse transcription polymerase reaction (RT-PCR) and PCR. 

### 2.3. Conventional PCR for Adenovirus Detection

The conventional PCR targeting hexon gene was used for the detection of MastAdV and AtAdV. The primers were used from the previously published literature [11]. The PCR reaction consisted of 5 µL of viral DNA, 1 µL of both forward and reverse primers, 0.5 µL dNTPs, 1.5 µL of 10× buffer, 0.75 µL of 50 mM MgCl_2_, 0.5 µL *Taq* Polymerase enzyme (Invitrogen, Waltham, MA, USA) and volume made to 25 µL with nuclease-free water (NFW). The nucleic acid from the positive sample confirmed by sequencing was used as positive control and NFW was used as negative control in all the PCR reactions. The thermocycling was carried out with an initial denaturation of 94 °C for 4 min; thereafter, 45 cycles of 94 °C at 1 min, 50 °C at 1 min, 72 °C at 2 min and final extension for 7 min, respectively. The positive samples were visualized onto 1.5% agarose gel and observed by the Gel Documentation system (Bio-Rad, Hercules, CA, USA). 

### 2.4. One-Step RT-PCR for Astrovirus Detection

The one-step RT-PCR used primers from a previously published panAstV assay, which amplifies a 420 bp fragment of the RdRp region [12]. A 5 μL of snap chilled RNA was added in 20 μL of reaction mixture containing 3.5 μL of NFW, 12.5 μL of 1× reaction buffer, 1 μL of SuperScript III RT/Platinum *Taq* Polymerase mix (Invitrogen), 1 μL each of forward and reverse primer. The RNA was denatured at 95 °C for 5 min, then immediately followed by a snap chill at 4 °C for 5 min. After that cDNA was synthesized at 42 °C for 45 min, then denaturation at 95 °C for 3 min, followed by 40 cycles of 94 °C for 15 s, 50 °C for 30 s, 68 °C for 2 min, final extension step was performed at 68 °C for 7 min, respectively. The positive samples were visualized as mentioned in Section 2.3. 

### 2.5. Sequencing and Phylogenetic Analysis

The purified PCR amplicons were subjected to cycle sequencing using Big Dye Terminator cycle sequencing kit v 3.1 (Applied Biosystems, Vilnius, Lithuania), and the dye terminators from the reaction were cleared with DyeEx 2.0 Spin Kit (Qiagen, Hilden, Germany). After post-purification, the sequencing was performed on an automated Genetic Analyzer ABI-PRISM 3720 (Applied Biosystems, Waltham, MA, USA). The chromatograms were analysed in Sequencing Analysis 5.2.0 (Applied Biosystems, Waltham, MA, USA). The BLAST was used for sequence similarity assessment. The study sequences, representative sequences of AstV and AdV genotypes, cognate gene sequences exhibiting maximum identity in BLAST, and Indian sequences were aligned using CLUSTALW with the MEGAv6.06 programme. The Neighbour-joining phylogenetic trees were constructed using the Kimura-2 parameter method, and 1000 bootstraps were used to estimate the confidence of the branch. The nucleotide identity was estimated using the same software by pairwise distance using the p-distance model. The AstV and AdV groups/genotypes were assigned to BAstV [13], PAstV [14], CAstV [15], PAdV [16] sequences, according to published reports. 

## 3. Results 

A total of 193 samples were collected from young animals, consisting of 48 samples from piglets (diarrheic 28; asymptomatic 20), 80 from pups (diarrheic 55, asymptomatic 25), and 65 from calves (55 diarrheic, 10 asymptomatic). In the rotavirus group A (RVA) screening, bovine (29.23%), and porcine (13.79%) samples were positive while all canine samples were negative (Table 1).

### 3.1. Adenovirus Detection and Phylogenetic Analysis

**Figure 1:**
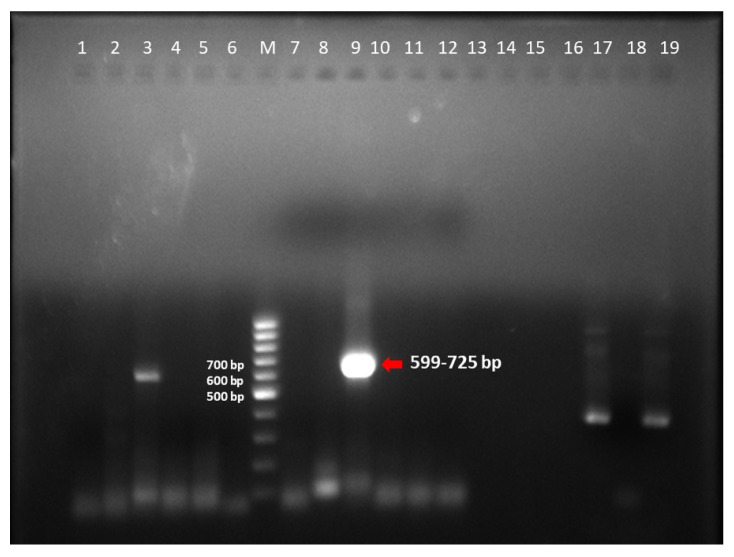
Representative Gel image of hexon gene amplified by PCR for the detection of porcine Adenovirus: Positive samples—Lane 9; Negative samples—Lane 4–6, 7, 8, 10–19: Lane M: 100 bp Ladder; Negative control—Lane 1 & 2; Positive control—Lane 3.

**Figure 2:**
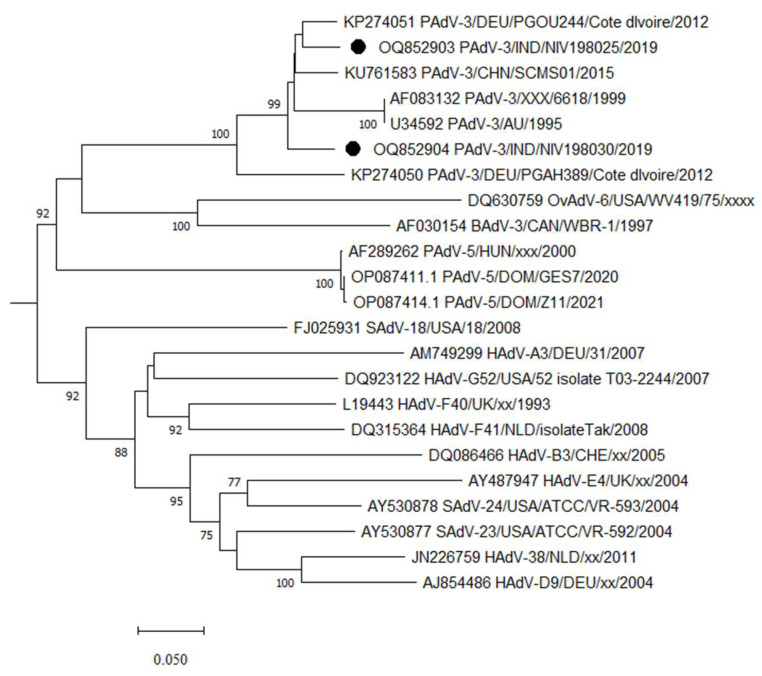
Phylogenetic analysis of hexon gene nucleotide sequences of adenoviruses detected from the porcine stool and faecal samples. The sequences detected in the present study are marked with a black solid circle. The AdV types were assigned to PAdV according to the published report [16].

Out of 48 porcine stool samples, two (4.1%) were positive for PAdV, while canine and bovine samples were negative for CAdV and BAdV. The size of hexon gene amplicons was variable in the range of 599–725 bp (Figure 1). The partial hexon gene of detected strains shared 93.4% of identity between themselves. The PAdV study strain exhibited 91.9–92.5% identity with PAdV-3 strain PGOU244/Cote d’Ivoire/2012 and Chinese PAdv-SCMS01 (Figure 2). However, the study strains exhibited 64.8–66% identity with PAdV-5 strains. The curated sequences were deposited in GenBank (OQ852903 and OQ852904).

### 3.2. Astrovirus Detection and Phylogenetic Analysis

**Figure 3:**
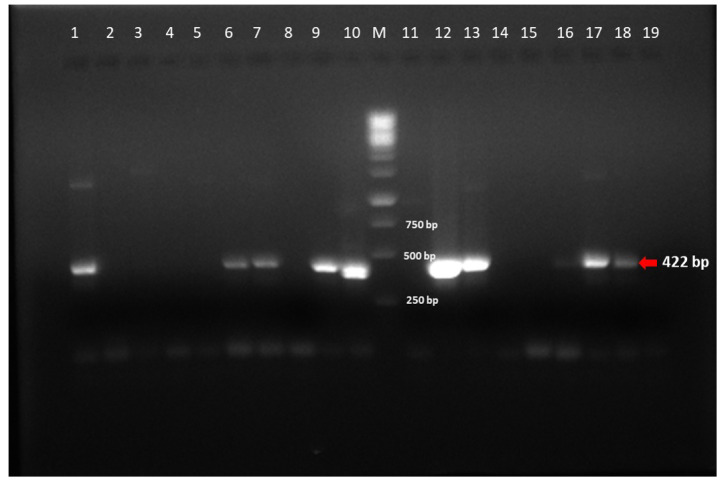
Representative Gel image of RdRp gene amplified by RT-PCR for the detection of porcine Astrovirus: Positive sample—Lane 6, 7, 9, 10, 12, 13, 17, 18; Negative sample—Lane 3–5, 8, 11, 14–16; Lane M: 1 Kbp; Positive control—Lane 1; Negative control—Lane 2.

**Figure 4:**
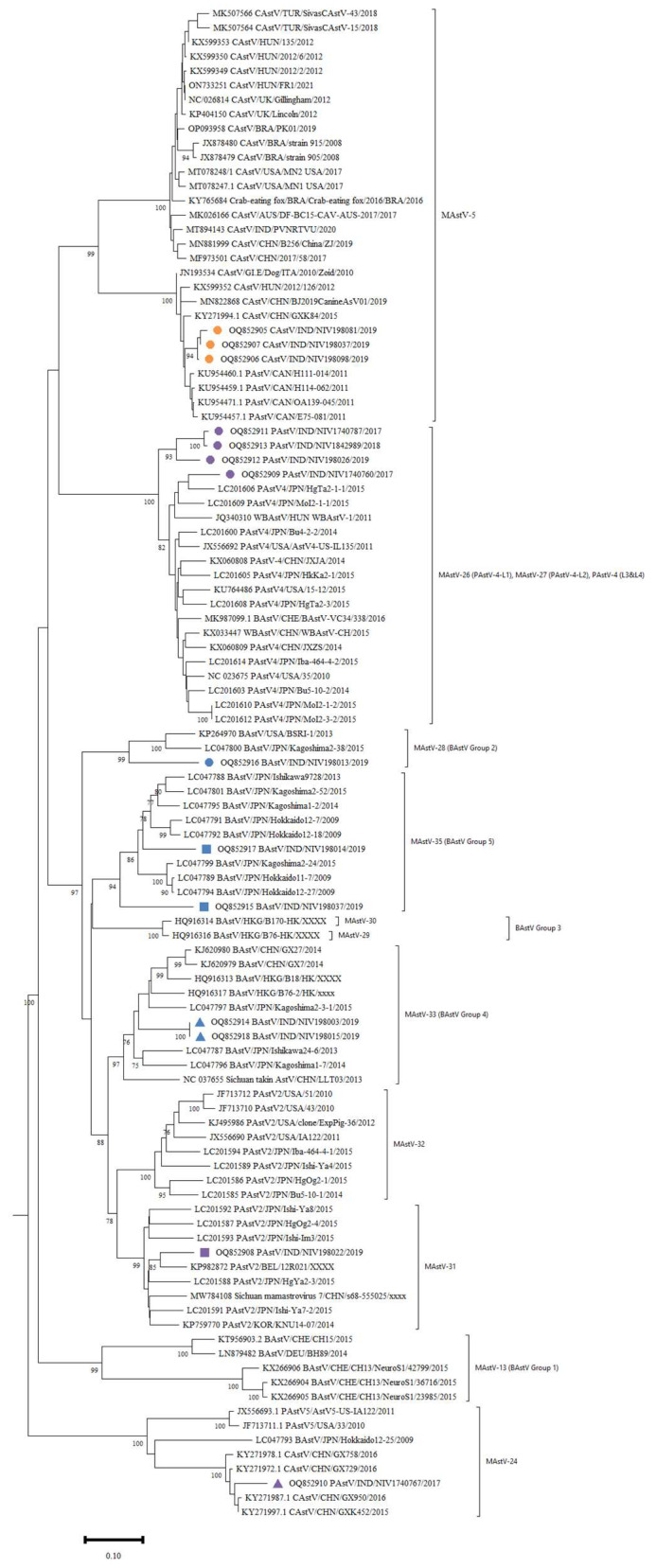
Phylogenetic analysis of RdRp nucleotide sequences of astroviruses detected from porcine, canine, and bovine stool and faecal samples. The AstV groups/genotypes were assigned to BAstV, PAstV and CAstV according to the published reports [13,14,15], respectively. The sequences detected in the present study are marked with coloured shapes.

The RdRp gene amplicons of AstV were of 422 bp size (Figure 3). The RdRp region nucleotide sequences of six PAstV, five BAstV and three CAstV study strains were compared with reference sequences from GenBank. The PAstV strains (NIV1740760, NIV1740787, NIV198026, and NIV1842989) displayed 88.1% identity with Chinese PAstV4 wild boar strain WBAstV-CH (MAstV-26) (Figure 4). For the remaining two study strains, NIV198022 showed 90.3% identity with PAstV2 strain Bel-12R021 (MAstV-31), and NIV1740767 showed 76.3–76.6% identity with American PAstV5 (MAstV-24) strains AstV5-US-IA122 and USA/33. The detected CAstV strains, NIV198081, NIV198098, and NIV198037 were identical and exhibited maximum identity in the 93.7–95.4% range with Chinese BJ2019CanineAsV01 and HUN/2012/126 (MAstV-5). All the detected BAstV strains shared nucleotide identity between 66.3–71%. The NIV198003 and NIV198015 (G4) were identical and displayed 86.2% identity with JPN/Kagoshima2-3-1 (MAstV-33), and study strain NIV198014 (G5) exhibited 87.7–87.4% identity with BoAstV/JPN/Hokkaido12-18 and BoAstV/JPN/Hokkaido12-27 (MAstV-35), study strain NIV198037 (G5) with (80.5%) BoAstV/JPN/Kagoshima2-24 and BoAstV/JPN/Hokkaido12-27 (MAstV-35), NIV198013 (G2) (79.7%) with USA/BSRI-1 and BoAstV/JPN/Kagoshima2-38 (MAstV-28). All the curated sequences were deposited in GenBank with accession numbers (OQ852905-OQ852918).

## 4. Discussion

The detection of AstVs and AdV in the gastrointestinal tract of both asymptomatic and diarrheic animals indicates that they are capable of causing persistent infections, coinfections or part of gut virome. Additionally, they have high genetic diversity, broad tissue tropism and disease spectrum, and form recombinants, making them potential emerging zoonotic viruses [17,18]. Therefore, detecting and characterizing gut pathogens like AstVs and AdVs with uncertain disease outcomes in animals is imperative. In this context, the present study on screening dog, pig and bovine samples revealed 12.5% of PAstV, 7.69% of BAstV, 3.75% of CAstV and 4.1% of PAdV, along with coinfections in piglets.

The PAdVs are suspected to be widespread in pigs, and among five PAdVs, PAdV3 is the most prevalent and well-characterized serotype [19]; however, their detection has not been reported from pigs reared in India. The present study identified and retrieved a total of two adenovirus sequences from piglets. After analysis of the partial hexon gene, the study strains found similarity with PAdV-3 reported from Africa (Cote d’Ivoire) and Asia (China) with 91.9–92.5% nucleotide identity. Our study reports the first detection of PAdV-3 from the Indian pig population, indicating that the PAdV3 circulating in Asia and Africa are genetically similar. The properties of PAdV-3 like genetic and structural similarities with HAdV-5, no cross-neutralization with Anti-HAdV-5 antibodies and transduction of human cells, imply that the development of PAdV-3 vectors will be a supplement to HAdV-5 vectors [19]. 

Five genotypes of PAstV (PAstV1-5) have been identified with varying prevalences from both healthy and diarrheic pigs, including the Czech Republic, Canada, Columbia, USA, Hungary, China, South Korea and Thailand [2,20,21]. The prevalence of PAstV (10.41%) in the present study was lower as compared to all published reports irrespective of the RT-PCR method used, except one Chinese study that reported a 2.82% positivity employing sequence-independent-single primer amplification (SISPA) [2,22]. The knowledge of PAstV diversity in Indian pigs is limited and only two studies have reported genotypes [23,24]. One study revealed the circulation of PAstV1, PAstV2, PAstV4, and PAstV5 in Haryana state with a predominance of the PAstV1 genotype. The second study covering a wider geographic area reported a predominance of PAstV4 and the detection of PAstV2 genotypes. The detected PAstVs belonged to PAstV2, PAstV4, and PAstV5. One study strain clustered with American PAstV5 strains, including BoAstV/JPN/Hokkaido12-25/2009 (LC047793.1), was believed to be derived from cross-species transmission [25]. 

Canine AstV has been reported to be associated with acute gastroenteritis or without clinical symptoms across the world. The only recently published study from the Hyderabad region of India reported a 7.2% prevalence of CAstV, and the strain with full genome sequence grouped with Chinese and Brazilian strains [26]. In contrast, all the present study strains are grouped with a different cluster of Hungarian, Italian and Chinese strains. The studied strains were from stray dogs and abandoned foreign dog breeds maintained in shelters in Pune city. Hence, the circulation of CAstVs resembling Hungarian, Italian and Chinese strains is quite possibly because of the ingestion of faecal material from foreign breeds. This suggests that there is a higher diversity of CAstV in India.

Most of the BAstVs detected in faecal samples from North and South America, Asia, Europe & African regions (Egypt) fall into G1, G2, G3, G4 and G5 groups [6]. The sequencing of the partial RdRp gene in the present study revealed the presence of BastV from divergent lineages G2 (MastV-28), G4 (MastV-33), and G5 (MastV-35). In the phylogenetic tree, all but one strain was clustered with Japanese bovine strains. The NIV198013 grouped with American and Japanese bovine strains. Importantly, no study strain found clustering with strains reported from neurological disease. The present study reported a much lower prevalence, which is in concurrence with the Korean and the United Kingdom study [27,28]. Nevertheless, to our knowledge, the present study reports the first-time detection of bovine AstVs from India. The majority of AstV sequences are lengthy fragments of 300–400 bp, which are not appropriate to study cross-species transmission and recombination, hence, complete genome sequencing is suggested to study the evolution of AstVs. 

The major viruses causing neonatal diarrhoea in bovines are RVA and bovine viral diarrhoea virus; in porcine are RVA, porcine epidemic diarrhoea virus and transmissible gastroenteritis virus; and in canines are parvovirus type 2, coronavirus, and canine distemper virus [1,2,26]. The majority of samples detected in this study were negative, indicating that the animals might be infected with major viruses, bacteria or parasites. The bovine (29.23%) and porcine (13.79%) samples were positive for RVA while all canine samples were negative. Additionally, the coinfection of RVA and AstV is in concurrence with previous studies reporting coinfection in piglets with multiple enteric pathogens [21]. The AstV and AdV coinfection has been reported in humans but we could not find such reports in pigs [29]. The results suggest that coinfections should be investigated in epidemiological studies, as they complicate the pathogenesis, diagnosis and prevention of enteric pathogens.

In conclusion, the study found AstVs circulating in puppies, piglets and calves with significant genetic diversity. However, AdV was detected only in piglets and co-infected with AstV with identity matching with strains from diverse geographical regions. The scarcity of genetic data on AstVs and AdV from the Indian animal population challenge laboratory diagnosis and prophylaxis strategies. In the future, large-scale surveillance targeting astrovirus and adenovirus should be initiated to understand genetic diversity, its contribution to disease, and the eventual exploitation of adenovirus as a xenogeneic vector for gene therapy and vaccine delivery.

## Figures and Tables

**Table 1:** The number of samples collected from apparently healthy and diarrheic porcine, bovine and canine species positive for RVA, AstV and AdV.

Species	Location	Health Condition	Sample Numbers	Virus Detected
Porcine (*n* = 48)	Chandkhed (22)	Diarrheic (18)	NIV1740787	AstV, RVA
			NIV1842989	AstV
			NIV1740785, NIV1740786, NIV1740788	RVA
		Apparently healthy (4)	Negative for RVA, AstV, AdV	
	Nandur (7)	Diarrheic (5)	NIV198025	AstV, AdV
			NIV198030	AstV, AdV
		Apparently healthy (2)	NIV198026	AstV
	Shirwal (19)	Diarrheic (5)	Negative for RVA, AstV, AdV	
		Apparently healthy (14)		
Bovine (*n* = 65)	Manchar (48)	Diarrheic (41)	NIV1841887-890, NIV1841892-897, NIV1841899, NIV1841901, NIV1841904-907, NIV1841909	RVA
		Apparently healthy (7)	Negative for RVA, AstV, AdV	
	Tathwade (17)	Diarrheic (14)	NIV198003, NIV198013,NIV198015	AstV
			NIV198014	AstV, RVA
			NIV198004, NIV198011	RVA
		Apparently healthy (3)	NIV198007	AstV
Canine (*n* = 80)	Kasarsai	Diarrheic (55)	NIV198081, NIV198098	AstV
		Apparently healthy (25)	NIV198036	AstV

## Data Availability

The sequences generated in the study are deposited in NCBI GenBank.
